# Percutaneous Closure of a Left Ventricular Pseudoaneurysm With Endovascular Coil Embolization: A Case Report

**DOI:** 10.7759/cureus.92616

**Published:** 2025-09-18

**Authors:** Kaori Singh, Rahul Rulia, Amit K Chaurasia

**Affiliations:** 1 Department of Cardiology, Artemis Hospitals, Gurugram, IND; 2 Department of Cardiology, Pandit Bhagwat Dayal Sharma Post Graduate Institute of Medical Sciences (PGIMS), Rohtak, IND

**Keywords:** coil embolization, left ventricular pseudoaneurysm, left ventriculogram, myocardial infarction, transcatheter closure device

## Abstract

Left ventricular pseudoaneurysm is a rare but potentially life-threatening complication following myocardial infarction. Although the management is usually surgical, percutaneous closure using occluder devices or coil embolization has proven to be a promising, minimally invasive alternative in high-risk cases.

We describe a 77-year-old female who presented three weeks after an anterior myocardial infarction with new-onset decompensated heart failure. Multimodality imaging, including echocardiography, left ventriculogram, and contrast-enhanced computed tomography thorax, revealed a basal left ventricular pseudoaneurysm with an approximately 2 mm neck, located in proximity to the aortic root and left anterior descending artery. Given her advanced age, decompensated heart failure, and multiple comorbidities, she was deemed high surgical risk, and percutaneous coil embolization was planned. Despite the anatomically challenging location, successful closure was achieved using four endovascular coils (Axium Prime - Medtronic, Irvine, CA, USA and MicroPlex - MicroVention, Inc., Aliso Viejo, CA, USA), with complete closure confirmed on post-procedure imaging.

At two-week follow-up, the patient remained clinically stable, with improvement in symptoms. This case illustrates that coil embolization can be a feasible option in selected high-risk patients with complex anatomy. It also highlights the importance of multimodality imaging, heart team discussions, and extensive pre-procedural planning in achieving successful outcomes.

## Introduction

A left ventricular pseudoaneurysm is a rare mechanical complication of myocardial infarction, caused by rupture of the ventricular free wall, contained by structures such as organized thrombus, adhesions, or the pericardium [1].

It usually develops subacutely after myocardial infarction [1] and is asymptomatic in nearly half of cases. When symptomatic, it may present with heart failure (15%), chest pain (13%), syncope or arrhythmias (10%), or systemic embolism (6%) [2]. Posterior pseudoaneurysms are over twice as common as anterior ones, likely because anterior wall rupture more often results in hemopericardium and sudden death [3].

Definitively diagnosing a pseudoaneurysm, distinguishing it from a true aneurysm, and defining its size and location can be difficult. This requires multimodality imaging to overcome the limitations of individual modalities.

Untreated subacute pseudoaneurysms carry a rupture risk of up to 48%, which makes a high index of clinical suspicion, timely diagnosis, and prompt, appropriate intervention essential. Surgical repair remains the standard treatment, but percutaneous intervention may be an option in selected high-surgical-risk patients. In our patient, advanced age, comorbidities, decompensated heart failure, and the anatomically challenging location of the lesion made percutaneous coil embolization a safer alternative to surgery.

We present the case of a 77-year-old female with a basal anterior left ventricular pseudoaneurysm after a recent anterior wall myocardial infarction. The lesion was located near critical structures, including the left anterior descending artery (LAD) and right aortic sinus, and was successfully treated with endovascular coil embolization.

## Case presentation

A 77-year-old female with a long-standing history of diabetes and hypertension for over two decades presented to our hospital with complaints of new-onset shortness of breath for two days. The dyspnea was present at rest and worsened on lying down. Three weeks prior, she had been hospitalized at another center with a week-long history of intermittent, severe chest pain. She was diagnosed with an anterior wall myocardial infarction with delayed presentation and underwent percutaneous coronary intervention, with deployment of two drug-eluting stents in the LAD. Echocardiography done during this admission showed akinesia involving the LAD distribution and a left ventricular ejection fraction of 40%.

**Figure 1:**
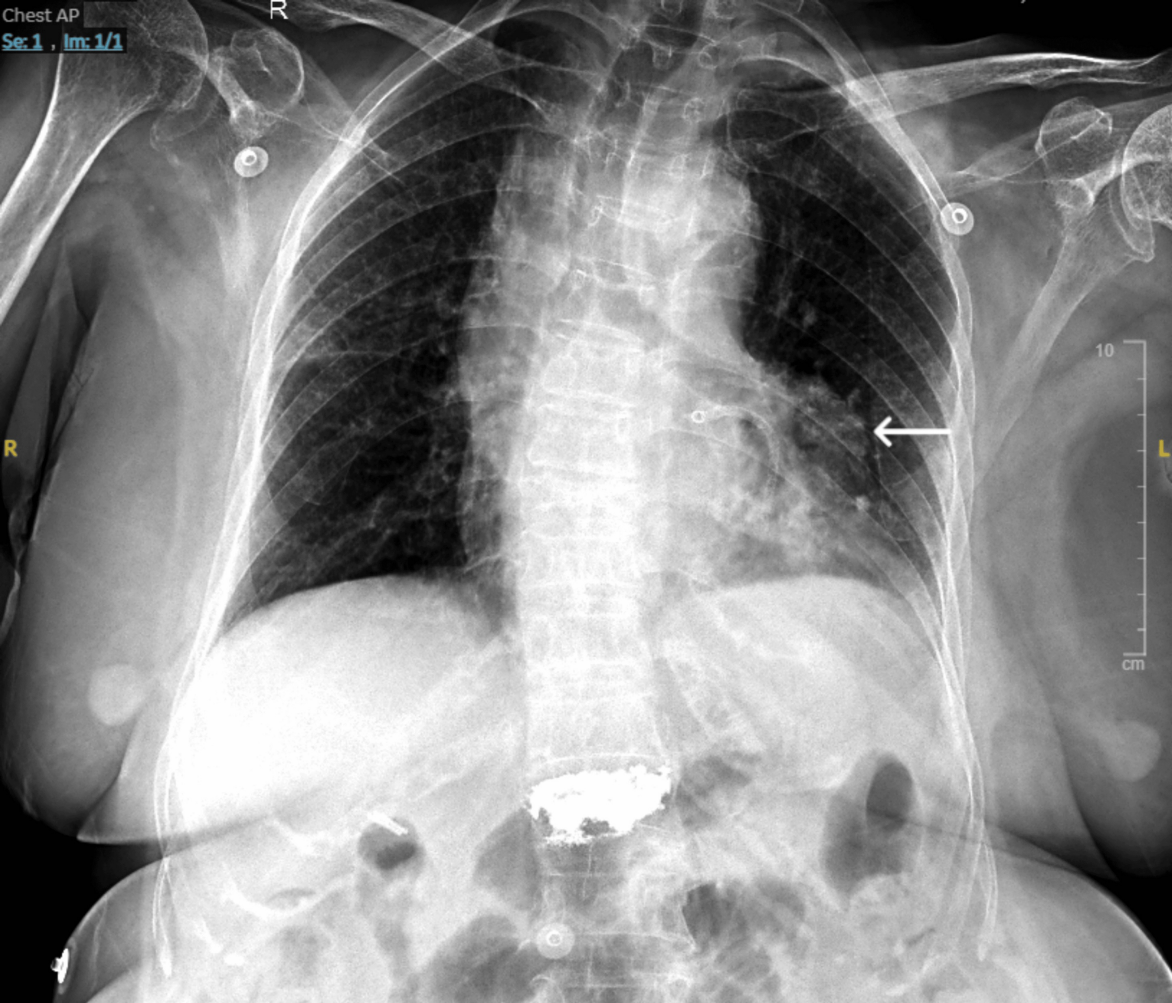
Chest X-ray (AP view) with an arrow highlighting the well-defined opacity adjacent to the upper border of the left ventricle. Note the shadow of the metallic stent present in the LAD. LAD: Left Anterior Descending Artery

At presentation to our centre, the patient was dyspneic yet conscious, hemodynamically stable, and maintaining an oxygen saturation of 97% on room air. Physical examination showed bilateral pitting pedal edema extending to the mid-calf and moderately elevated jugular venous pressure. Auscultation revealed fine crepitations in the bilateral basal lung fields, without any audible murmur. Her electrocardiogram (ECG) showed nonspecific ST-T wave changes, with no new abnormalities compared to previous tracings. Chest X-ray showed a well-defined opacity adjacent to the silhouette of the upper left ventricle (LV) (Figure 1).

Laboratory investigations revealed a markedly elevated NT-proBNP level of 10,452 pg/mL, consistent with significant heart failure. High-sensitivity troponin I was elevated at 3,745 pg/mL, despite no supportive clinical or electrocardiographic evidence of ongoing ischemia. Serial measurements showed a downward trend. Complete blood count, renal function tests, electrolytes, and coagulation parameters were within normal limits.

In view of new-onset heart failure in the setting of a recent myocardial infarction, initial 2D transthoracic echocardiography (TTE) was performed, revealing a thin-walled, non-contractile cavity with a narrow neck arising from the basal segment of the LV, measuring approximately 6 mm in diameter. Doppler imaging demonstrated bidirectional turbulent flow within the cavity (Video 1). There was no evidence of any associated thrombus. Although these echocardiographic findings were highly suggestive of a pseudoaneurysm, precise localization and sizing were limited by suboptimal acoustic windows, secondary to the patient’s body habitus, as well as the need for unconventional imaging planes to visualize the cavity.

**Video 1:** TTE with Doppler showing a pseudoaneurysm arising from the free wall of the basal LV, measuring approximately 6 mm in diameter. Doppler study shows a to-and-fro flow through a narrow neck. TTE: Transthoracic Echocardiography; LV: Left Ventricle

Following this, a heart team meeting was organized to decide on the best course of management. She was deemed high risk for surgical intervention due to acute decompensated heart failure, ongoing dyspnea, and advanced age. After a thorough discussion with the patient and the team, a decision to proceed with percutaneous closure was made over surgical closure. A left ventriculogram was planned, following which the approach for percutaneous closure would be evaluated.

The left ventriculogram was performed via right femoral artery access. It confirmed the findings of the echocardiogram, demonstrating a pseudoaneurysm arising from the basal segment of the LV, in close proximity to the right aortic sinus and the proximal LAD (Video 2). A targeted contrast injection into the pseudoaneurysm revealed an extremely narrow neck, measuring approximately 2 mm. The pseudoaneurysm cavity measured approximately 2 cm in maximum diameter, significantly larger than estimated on TTE, reflecting differences in resolution and imaging planes of the two modalities (Video 3). An aortic root angiogram was performed, which ruled out any communication between the pseudoaneurysm and the aortic sinuses or coronary arteries, and confirmed good flow through the coronaries, including the previously stented LAD. 

**Video 2:** Left ventriculogram showing the pseudoaneurysm (A) over the basal LV. Note the proximity of the LAD (B), and the right aortic sinus (C). LAD: Left Anterior Descending Artery; LV: Left Ventricle

**Video 3:** Selective contrast injection into the pseudoaneurysm cavity shows an extremely narrow neck, approximately 2 mm in diameter, opening into a well-defined pseudoaneurysm cavity measuring 2 cm in maximum diameter.

Planning for percutaneous closure presented two main challenges: ensuring secure access to the pseudoaneurysm cavity through its narrow neck, and protecting nearby high-risk structures, such as the proximal LAD and right aortic sinus. These anatomical constraints made the use of bulkier closure devices, such as septal occluders or vascular plugs, less suitable. Instead, endovascular coils were selected due to their success in small- to medium-sized pseudoaneurysms, ease of navigation through narrow necks, and lower risk of mechanical injury. While this method carried a higher risk of incomplete closure, it was considered the safest and most feasible option, given the location and morphology of the pseudoaneurysm.

The closure was initiated via a retrograde trans-arterial approach through the left femoral artery. After accessing the LV, selective cannulation of the pseudoaneurysm neck was performed using a 7F EBU guide catheter (Medtronic, Irvine, CA, USA), through which a Fielder FC guidewire (Asahi Intecc Co., Ltd., Aichi, Japan) was passed into the pseudoaneurysm. Following this, a Headway 17 microcatheter (MicroVention, Inc., Aliso Viejo, CA, USA) was taken over the guidewire and introduced into the cavity. Through the microcatheter, four detachable endovascular coils were deployed into the cavity of the pseudoaneurysm (Axium Prime 10 x 30 mm - Medtronic, Irvine, CA, USA and MicroPlex 18 x 59 mm, 16 x 52 mm, and 20 x 65 mm - MicroVention, Inc., Aliso Viejo, CA, USA). Post-deployment contrast injection demonstrated markedly reduced flow into the cavity, indicating successful embolization (Video 4), which was confirmed by intraprocedural transesophageal echocardiography (TEE). Aortic root and coronary angiogram done post-coil embolization showed good flow through the LAD and no evident valvular complications. The femoral access site was closed using one ProGlide (Abbott Vascular, Santa Clara, CA, USA), and the patient was shifted for post-procedural care in a stable condition.

**Video 4:** Post-procedure contrast injection showing a markedly reduced flow into the pseudoaneurysm cavity. Metallic coils are seen in the cavity.

**Figure 2:**
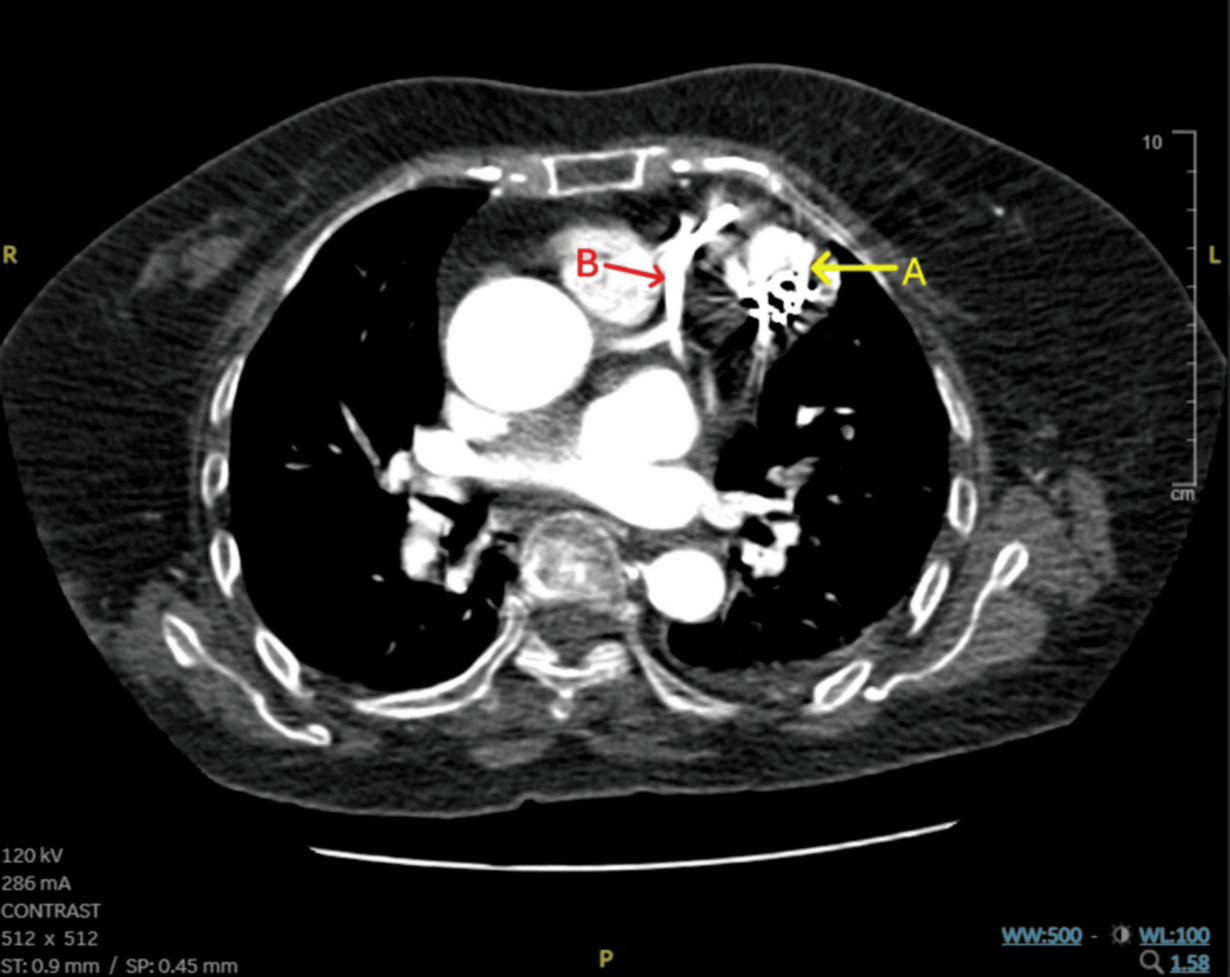
Contrast-enhanced CT thorax image. Contrast-enhanced CT thorax shows a pseudoaneurysm measuring 18 x 23 mm², located in the anterior segment of the LV (A), containing metallic coils. It is situated lateral to the LAD, with no visible communication between them (B). LAD: Left Anterior Descending artery; LV: Left Ventricle; CT: Computed Tomography

Post-procedure TTE showed metallic coils in situ, with no evidence of residual flow into the pseudoaneurysm cavity (Video 5). To better delineate its anatomy and assess procedural outcome, a contrast-enhanced computed tomography (CT) of the thorax was performed. It revealed a well-defined, rounded cavity with a narrow neck arising from the anterior segment of the basal LV, situated lateral to the course of the LAD. The maximum diameter was 23 mm, the dome height was 18 mm, and the neck width was found to be 10 mm post-coil embolization. The deployed coils were visible within the neck of the pseudoaneurysm, extending into the cavity and producing a localized metallic artifact (Figure 2). There was no evidence of active contrast extravasation, coil migration, or residual flow within the pseudoaneurysm cavity - findings consistent with effective occlusion without any procedural complications. Measurements on CT varied from pre-procedure left ventriculogram values due to both the presence of coils within the neck and cavity, and differences in the imaging modality used. 

**Video 5:** Post procedure TTE shows a metallic coil shadow within the pseudoaneurysm cavity. No residual flow into the pseudoaneurysm cavity is seen on the Doppler study. TTE: Transthoracic Echocardiography

She recovered well following the procedure and was discharged two days later. She was continued on dual antiplatelet therapy for her recent myocardial infarction throughout admission and at discharge, alongside standard heart failure medications. At her one-week follow-up, she was asymptomatic with improved effort tolerance. Repeat TTE at 14 days demonstrated an ejection fraction of 40%-45%, with no significant residual flow within the pseudoaneurysm cavity. These findings suggest that the procedure was feasible, well-tolerated, and resulted in sustained occlusion with early clinical improvement. Unfortunately, long-term follow-up was not possible, as the patient developed fatal pneumonia and sepsis 1.5 months after the procedure.

## Discussion

A left ventricular pseudoaneurysm occurs when a cardiac free wall rupture is contained by adherent pericardium, organized thrombus, or fibrous scar tissue rather than myocardium [4]. The true incidence of left ventricular pseudoaneurysm remains poorly defined, with a recent review estimating it at approximately 0.2% to 0.5% [5]. Lacking the structural reinforcement of myocardial tissue, these lesions carry a high risk of rupture, reportedly ranging from 30% to 45% [3]. Due to its rarity, non-specific presentation, and diagnostic challenges, LV pseudoaneurysm can be missed, resulting in potentially fatal consequences. This necessitates a high degree of clinical suspicion for its timely diagnosis and prompt management.

TTE with color Doppler is a key initial diagnostic tool for suspected LV pseudoaneurysm, showing some abnormality in 85%-90% of patients but confirming a definitive diagnosis in just 29% of patients [3]. Moreover, its wide availability and low cost reinforce its role as a screening test. Early studies suggested that the echocardiographic diagnostic criteria of a neck-to-cavity diameter ratio of ≤0.5 can be used to diagnose pseudoaneurysms and differentiate them from true aneurysms [6]. While these features were present in our case, further studies report that up to 20% of pseudoaneurysms do not meet these criteria [3]. Echocardiography is also limited by the quality of acoustic windows achieved and the need for unconventional imaging views to visualize difficult areas [3,6]. As a result, TTE alone is not sufficient to form a definitive diagnosis in most cases, and further imaging is usually warranted.

Left ventriculography has traditionally been considered the ‘gold standard’ for diagnosing left ventricular pseudoaneurysms [7] and can establish a definitive diagnosis in up to 87% of cases [3]. Key angiographic features include a saccular, akinetic outpouching with a narrow neck arising from the LV and absence of communication with the coronary arteries [3,5]. In addition to accurately delineating the size and anatomical location of the lesion, ventriculography also enables assessment of coronary artery disease, which is crucial for procedural planning [5]. Although valuable, contrast injection and catheter manipulation during ventriculography carry a risk of infection, rupture in large pseudoaneurysms, and embolization in the presence of thrombus - complications that may be fatal [5]. Ventriculography should therefore be used with caution or avoided in select cases. In our case, ventriculography and selective contrast injection successfully localized the pseudoaneurysm at the basal LV, adjacent to the right aortic sinus and LAD, and confirmed the size to be larger than estimated by echocardiography. Aortic root angiography showed no communication with the coronary arteries or the aortic root. This comprehensive anatomical assessment was essential in confirming the diagnosis and informing the subsequent treatment strategy.

While echocardiography and left ventriculography remain key diagnostic tools, non-invasive modalities such as cardiac CT and magnetic resonance imaging (MRI) are increasingly being used to improve diagnostic accuracy. Independently, CT and MRI have been seen to form a definitive diagnosis in 7% and 53% of cases, respectively. However, when used to supplement the findings of ventriculography, improved diagnostic accuracy is seen in 42% of patients who also underwent a CT, and 80% of patients who also underwent an MRI [3]. Cardiac CT provides a rapid 3D image of the pseudoaneurysm, along with concurrent coronary assessment [8]. High-resolution CT angiography with 3D reconstruction [9] and ECG-gated multidetector CT [10] have also been used for enhanced anatomical visualization. Cardiac MRI offers higher spatial resolution and superior tissue characterization, enabling assessment of myocardial viability and differentiation of pseudoaneurysms from true aneurysms [8,9]. However, its use may be limited in orthopneic or hemodynamically unstable patients due to longer acquisition times, which is why our patient was unable to tolerate the scan [9]. Ultimately, accurate diagnosis of a left ventricular pseudoaneurysm requires multimodality imaging to define its anatomy comprehensively and guide management, and there are various options to choose from.

Conservative medical management of an LV pseudoaneurysm has a poor prognosis, with a mortality rate of 48% [3]. Surgical closure is considered the treatment of choice; however, it can be associated with significant operative risk, with estimated mortality rates ranging from 10% to 23% [3]. Percutaneous closure offers a promising, minimally invasive alternative, particularly in surgically high-risk patients such as ours. However, its successful implementation requires a detailed understanding of the pseudoaneurysm’s anatomy, including its spatial relationship to adjacent coronary arteries and valvular structures [11].

The two main percutaneous closure techniques currently used include endovascular coil embolization and placement of septal occluder devices, or a combination of both [11]. Coils offer advantages such as easier deployment, lower risk of mechanical complications, and suitability for small- to medium-sized lesions. However, they are associated with a higher risk of incomplete closure, particularly in larger pseudoaneurysms. In contrast, septal occluders are more likely to achieve complete closure but are larger in size and technically challenging due to stiff delivery systems. Due to their size, they may compress adjacent structures, limiting their use in difficult anatomies [11].

In our case, coil embolization was selected for the patient over septal occluders due to the small size of the false cavity, narrow neck of the pseudoaneurysm, and a high-risk location in proximity to the aortic root and LAD. The procedure involved deployment of four endovascular coils, following which intra-procedural imaging with selective contrast injection and TEE demonstrated a marked reduction in flow into the pseudoaneurysm cavity. The patient was on dual antiplatelet therapy for her recent myocardial infarction, which was continued peri-procedure. Post-procedure, both transthoracic Doppler echocardiography and contrast-enhanced CT confirmed complete closure, with no significant residual flow through the neck or any evidence of coil migration.

Although there is limited literature on long-term follow-up of coil embolization for LV pseudoaneurysm, a three-patient study on left ventricular outflow tract (LVOT) pseudoaneurysms showed no recurrence or reintervention for up to five years after successful coil embolization [12]. Established complications of coil embolization in other vascular pseudoaneurysms - including delayed recanalization, coil migration, or erosion - although theoretically possible, lack reported data in the context of LV pseudoaneurysms. 

While this case highlights the evolving role of percutaneous closure in the management of left ventricular pseudoaneurysms, it has several limitations. Our technique has limited generalizability due to the very rare and specific anatomy of the lesion. Moreover, comparison with existing literature is challenging, given the scarcity of published cases and the absence of well-established management guidelines. The lack of long-term follow-up in this case prevents us from commenting on the durability and recurrence risk after coil embolization.

Despite these shortcomings, to our knowledge, our case report is a first-of-its-kind case of a post-myocardial infarction left ventricular pseudoaneurysm in a rare anterolateral basal location, treated successfully with coil embolization. It underscores the importance of tailoring intervention to anatomical considerations and demonstrates the feasibility and safety of coil embolization in treating a high-risk pseudoaneurysm.

## Conclusions

Left ventricular pseudoaneurysm is a rare complication of myocardial infarction, with a high risk of life-threatening complications. Diagnosis is often difficult due to nonspecific presentations and limitations of individual imaging modalities, making a multimodal approach essential - using echocardiography, ventriculography, cardiac CT, or cardiac MRI, as appropriate. For patients at high surgical risk, percutaneous closure offers a minimally invasive alternative. Among available techniques, coil embolization is particularly suitable for small- to moderate-sized pseudoaneurysms, offering easier deployment, lower mechanical risk, and greater safety in anatomically challenging locations. Successful closure depends on careful pre-procedural imaging and appropriate case selection, including factors such as neck diameter, pseudoaneurysm size, and proximity to critical structures.

Our case demonstrates the safety and efficacy of coil embolization to treat a rare basal anterior pseudoaneurysm located near the aortic root and LAD. It highlights the emerging role of coil embolization in the percutaneous closure of left ventricular pseudoaneurysms, while underscoring the need for larger-scale studies and longer-term follow-up to better define durability and clinical outcomes.
